# Supplementary material for: Recombinant Antigen of Type 2 Porcine Reproductive and Respiratory Syndrome Virus (PRRSV-2) Promotes M1 Repolarization of Porcine Alveolar Macrophages and Th1 Type Response
Source: Vaccines (Basel). 2021 Sep 10;9(9):1009. doi: 10.3390/vaccines9091009 (PMC8473084; doi:10.3390/vaccines9091009)
Supplement: Supplementary file 1 [file vaccines-09-01009-s001.zip › vaccines-1288768-supplementary.pdf]

vaccines

Supplementary Materials

(a)

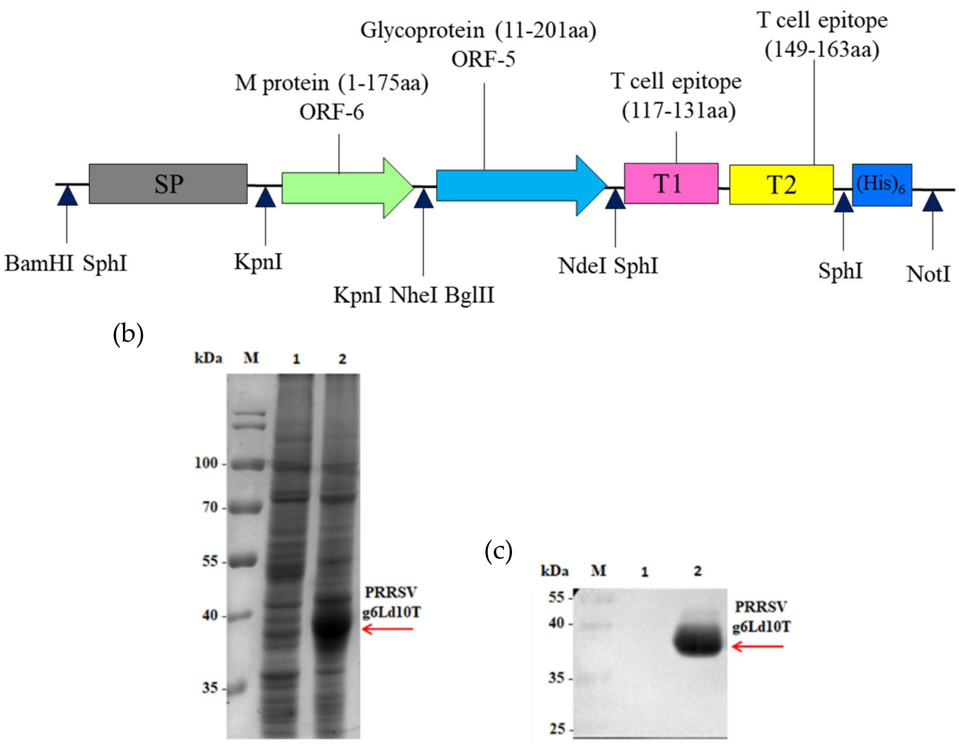

Figure S1. Production of recombinant g6Ld10T protein (A1) in baculovirus system . Schematic representation of the g6Ld10T transfer vector construct. (a) The coding sequence of the PRRSV gene and T-cell epitopes were cloned into baculovirus transfer plasmid between enzyme restriction sites to form the transfer vector. This transfer vector was co-transfected into Hi-5 insect cells along with the baculovirus to form recombinant baculovirus containing PRRSV-2 antigen. SDS-PAGE (b) and Western blot analysis (c) of recombinant g6Ld10T protein. lane M: protein marker; lane1: crude cell extract from Hi-5; lane 2: crude cell extract from Hi-5 infected with recombinant baculoviruses expressing g6Ld10T.

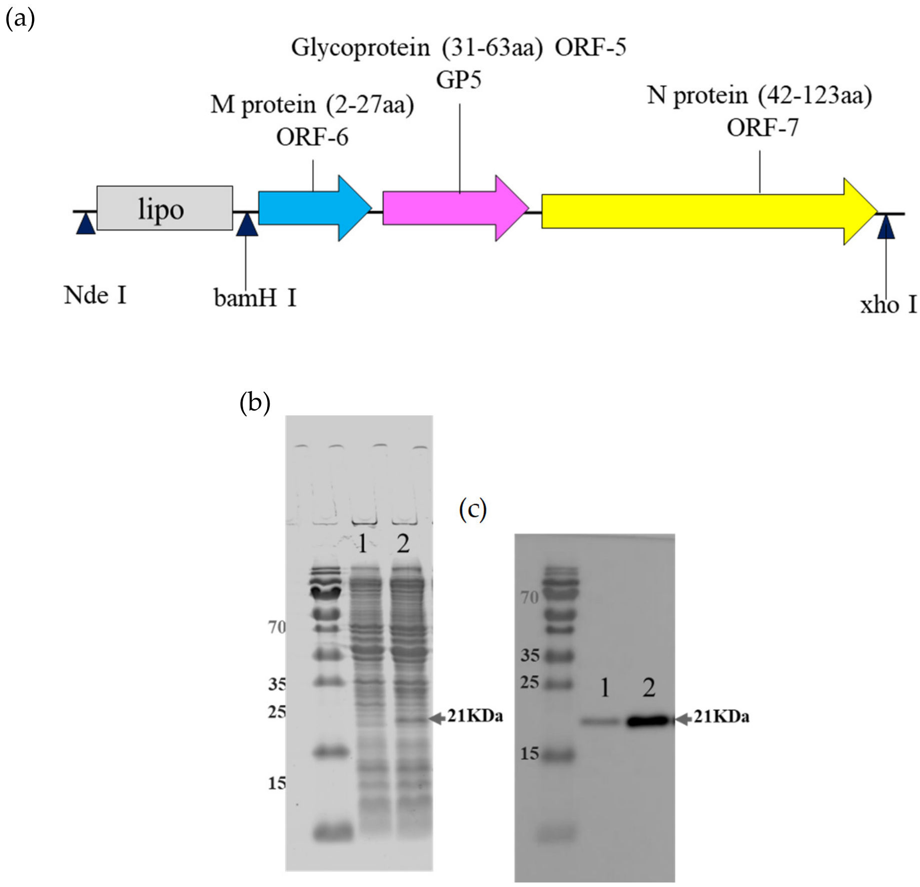

Figure S2. Production of recombinant lipo-M5Nt protein (A2) in *E. coli*. Schematic representation of the lipo-M5Nt transfer vector construct. (a) The polyacrylamide gel image of the purified recombinant lipo-M5Nt protein produced by the empty vector in *E. coli*. SDS-PAGE (b) and Western blot analysis (c) of recombinant lipo-M5Nt protein. lane M: protein marker; lane1: non protein induction; lane 2: induction of recombinant protein expression.

(a)

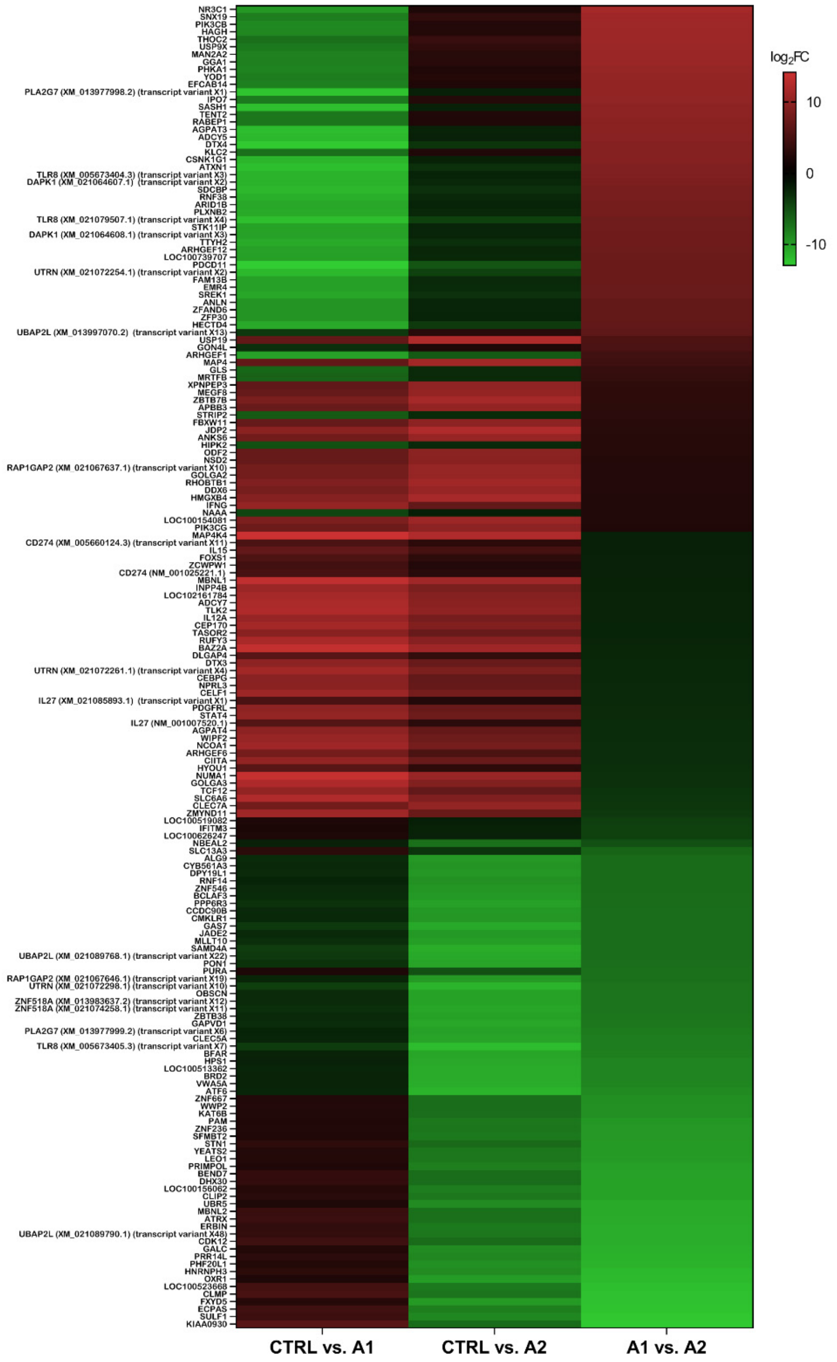
log2FC
10
0
-10
CTRL vs. A1
CTRL vs. A2
A1 vs. A2

(b)

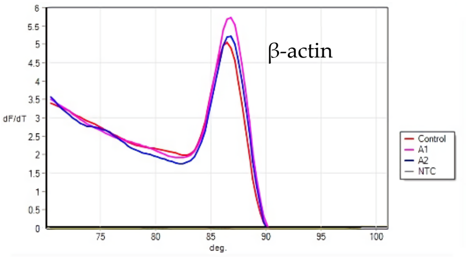
β-actin
dF/dT
deg.
Control
A1
A2
NTC

(c)

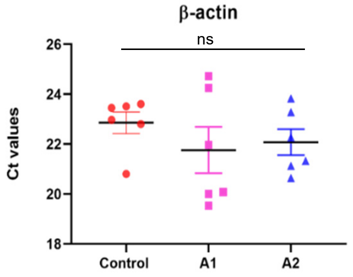
β-actin
ns
Ct values
Control
A1
A2

(d)

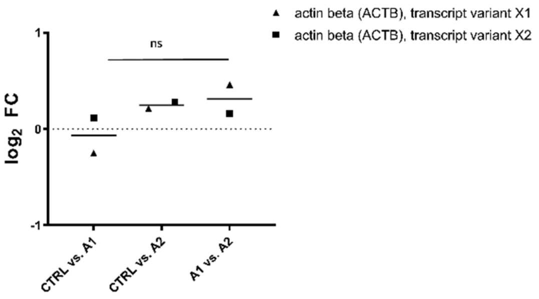
actin beta (ACTB), transcript variant X1
actin beta (ACTB), transcript variant X2
ns
log2 FC
CTRL vs. A1
CTRL vs. A2
A1 vs. A2

Figure S3: Trancriptomic analysis of the differential genes between the groups. (a) Heatmap of differential gene expression at each group comparison. The X-axis represents the comparison of each sample group and the Y-axis is the name of the 176 genes for each group. The color bar above the heatmap annotates the scores of gene expression level in the heatmap (b) Representative data of the melting curve of *β-actin* in RT-PCR. (c) The cycle threshold (Ct) values of *β-actin* for each group in RT-PCR. (d) Fold change in expression levels of *β-actin* for each comparison group was elucidated from NGS data. Data were calculated from the cDNA libraries collected by pooling of six samples in each group, n=6.
